# Cryptosporidiosis outbreaks linked to the public water supply in a military camp, France

**DOI:** 10.1371/journal.pntd.0010776

**Published:** 2022-09-12

**Authors:** Stéphanie Watier-Grillot, Damien Costa, Cédric Petit, Romy Razakandrainibe, Sébastien Larréché, Christelle Tong, Gwenaëlle Demont, David Billetorte, Damien Mouly, Didier Fontan, Guillaume Velut, Alexandra Le Corre, Jean-Christophe Beauvir, Audrey Mérens, Loïc Favennec, Vincent Pommier de Santi

**Affiliations:** 1 French Armed Forces Centre for Epidemiology and Public Health (CESPA), Marseille, France; 2 Rouen Normandy University, EA7510 ESCAPE, CNR Expert Laboratory for Cryptosporidiosis, Rouen, France; 3 French Armed Forces Health Service, France; 4 Bégin Military Teaching Hospital, Paris, France; 5 Regional Health Service of Occitanie, Territorial Delegation, Montauban, France; 6 Santé Publique France, Regional Office of Occitanie, Toulouse, France; 7 Departmental Laboratory 31, Launaguet, France; 8 Aix Marseille University, IRD, AP-HM, SSA, VITROME, Marseille, France; 9 IHU-Méditerranée Infection, Marseille, France; University of Oxford, UNITED KINGDOM

## Abstract

**Introduction:**

Contaminated drinking and recreational waters account for most of the reported *Cryptosporidium* spp. exposures in high-income countries. In June 2017, two successive cryptosporidiosis outbreaks occurred among service members in a military training camp located in Southwest France. Several other gastroenteritis outbreaks were previously reported in this camp, all among trainees in the days following their arrival, without any causative pathogen identification. Epidemiological, microbiological and environmental investigations were carried out to explain theses outbreaks.

**Material and methods:**

Syndromic diagnosis using multiplex PCR was used for stool testing. Water samples (100 L) were collected at 10 points of the drinking water installations and enumeration of *Cryptosporidium* oocysts performed. The identification of *Cryptosporidium* species was performed using real-time 18S SSU rRNA PCR and confirmed by GP60 sequencing.

**Results:**

A total of 100 human cases were reported with a global attack rate of 27.8%. *Cryptosporidium* spp. was identified in 93% of stool samples with syndromic multiplex PCR. The entire drinking water network was contaminated with *Cryptosporidium* spp. The highest level of contamination was found in groundwater and in the water leaving the treatment plant, with >1,000 oocysts per 100 L. The same *Cryptosporidium hominis* isolate subtype IbA10G2 was identified in patients’ stool and water samples. Several polluting activities were identified within the protection perimeters of the water resource. An additional ultrafiltration module was installed at the outlet of the water treatment plant. After several weeks, no *Cryptosporidium* oocysts were found in the public water supply.

**Conclusions:**

After successive and unexplained gastroenteritis outbreaks, this investigation confirmed a waterborne outbreak due to *Cryptosporidium hominis* subtype IbA10G2. Our study demonstrates the value of syndromic diagnosis for gastroenteritis outbreak investigation. Our results also highlight the importance of better assessing the microbiological risk associated with raw water and the need for sensitive and easy-to-implement tools for parasite detection.

## Author summary

Cryptosporidiosis remains a neglected infectious disease, even in high-income countries. Most of the reported cases and outbreaks are related to drinking water and recreational water contaminated with *Cryptosporidium* spp. In Europe, the search for *Cryptosporidium* spp. and other parasites in stool or water samples is not routinely performed by laboratories, especially in the absence of dedicated national guidance on testing. In France, cryptosporidiosis is not a notifiable disease. In order to better assess the pathogens involved in foodborne and waterborne disease outbreaks a new outbreak investigation strategy was implemented in the French Armed Forces including: systematic stool sampling, routine syndromic multiplex PCR diagnoses, and pathogens genotyping. After several unexplained gastroenteritis outbreaks in a military camp in France, we identified the same *C*. *hominis* IbA10G2 isolate in the stools of patients and in the entire water distribution network. The highest levels of contamination were found in groundwater and in the water leaving the treatment plant. Our study demonstrates the value of syndromic diagnosis for gastroenteritis outbreaks investigation and highlights the importance of better assessing the microbiological risks associated with raw water.

## Introduction

*Cryptosporidium* spp. is a widely distributed zoonotic protozoan that infects the epithelial cells of the gastrointestinal tract of vertebrates. Cryptosporidiosis is endemic worldwide, mostly affecting children under the age of five in low-income countries [1,2]. Two major species affect humans: *C*. *parvum* and *C*. *hominis* [1]. Cattle are major hosts for *C*. *parvum* [3,4]. The oocyst, the infectious stage of *Cryptosporidium*, can survive in water and soil for many months. Infected humans and animals contaminate the environment by shedding infective oocysts in their faeces. The oocysts are able to withstand standard water treatment and the concentrations of chlorine commonly used [5]. Transmission of *Cryptosporidium* spp. occurs mainly indirectly through ingestion of contaminated water (e.g. drinking or recreational water) or food (e.g., raw milk) or directly through person-to-person or animal-to-person routes [6,7]. A low infective dose, 10–30 oocysts can be sufficient to cause the disease in healthy persons [8]. The incubation period is an average of seven days (from 1 to 10 days) [1]. In immunocompetent patients, prolonged watery diarrhoea (>10 days) is commonly observed, associated with other symptoms such as nausea, abdominal cramps, low-grade fever or anorexia. These gastrointestinal symptoms usually resolve spontaneously within 2–3 weeks [9]. Symptoms can be severe and life-threatening for immunocompromised individuals as well as young infants. Persistence of intestinal symptoms, after resolution of the acute stage of illness, can occur and may be indicative of post infectious irritable bowel syndrome [10]. Both innate and adaptive immune responses are involved in clearing infection in human [11]. Repeated infections could lead to partial immunity against reinfection [12].

Contaminated drinking and recreational waters account for most of the reported *Cryptosporidium* spp. exposures in high-income countries. Waterborne outbreaks are reported each year in Europe and the United States [13]. Major outbreaks occurred in 1993 in Milwaukee, USA (400,000 cases), and in 2010 in Sweden (27,000 cases) [6,14,15]. In the European Union (EU) and the European Economic Area (EEA), cryptosporidiosis is a notifiable disease and surveillance data are collected through the European Surveillance System (TESSy) [13,16]. Seasonality is usually observed, with an incidence increase in April and September [13]. Notification of cryptosporidiosis is not mandatory in Austria, Denmark, France, Greece and Italy; thus, cryptosporidiosis data in Europe remain incomplete. In France, only foodborne and waterborne disease outbreaks (FBDOs) are subject to mandatory notification. *Cryptosporidium* spp. was involved in half of waterborne disease outbreaks investigated in France between 1998 and 2008 [17].

Cryptosporidiosis is not subject to mandatory surveillance in the French Armed Forces (FAF). On June 14th and 22nd, 2017, two successive outbreaks of acute gastroenteritis (AG) were reported to the FAF epidemiological surveillance system. They occurred in a military training camp in a rural area, near the municipality of Caylus (1,500 inhabitants), Southwest France. This camp regularly hosts groups of service members from other military units in France. These outbreaks affected two companies (A and B) of 180 individuals each, deployed from Northwest France (A then B). From January 2015 to February 2017, four other gastroenteritis outbreaks were reported in this camp, all among trainees in the days following their arrival. The previous investigations identified poor hygiene conditions during field activities as one possible explanation for these outbreaks but no causative pathogen. However, biological analyses of stools were limited to the detection of bacteria and viruses. The hypothesis of water contamination was ruled out considering baseline quality levels observed on drinking water analyses. We report the results of the epidemiological, microbiological and environmental investigations conducted in 2017.

## Material and methods

### Study design

In order to better assess the pathogens involved in FBDOs in the FAF, a rigorous investigation method was implemented in 2017 and applied in this study. This method was based on rapid investigations, secure human and environmental sampling, and sample analysis by expert laboratories. These investigations started as soon as a suspected FBDO was reported to the French military’s epidemiological surveillance system. This approach consisted of: i/ the collect of stool samples, immediately during primary care, from at least 5 of the most symptomatic patients; ii/ the transport of stool samples by an authorized carrier, in compliance with national and international regulations, to a military reference laboratory, for syndromic diagnosis using multiplex PCR; iii/ conducting a case-control epidemiological survey, in which the exposed military population is interviewed using a standardised questionnaire; iv/ carrying out environmental investigations by a multidisciplinary team deployed on site and including environmental sampling; v- the broad-spectrum testing of environmental samples for pathogens by reference laboratories; vi- the strain genotyping of pathogens found in stool and environmental samples by the National Reference Centre Expert Laboratory for the pathogen concerned.

### Epidemiological investigations

An extended search for AG cases was made in both companies using the following definition: “Patient with diarrhoea or vomiting or abdominal pain in June 2017, and present at the military camp during the 15 days prior to symptom onset”. In addition, a case-control survey was carried out among 101 members of Company B—60 cases and 41 controls—using a self-administered questionnaire, to identify an association between the disease and food or beverages consumption since their arrival.

The investigation was also extended to the local civilian population to identify a concurrent outbreak or preceding AG clusters over the period from 2015 to 2017. To this end, drug reimbursement data associated with AG treatment were extracted from the French National Health Insurance database and a space-time detection method for cluster detection applied, as described [18,19]. Rainfall data were obtained from the Météo France website (https://meteofrance.com/climat/france/montauban). Statistical analyses were performed using R Software, version 3.4.2.

### Microbiological investigations

Stool samples were collected from 14 patients, 11 and 3 in Company A and B respectively. Stool samples were collected by the medical unit of the military camp. They were stored at +2°C—+8°C until transport with cold chain monitoring to the Bégin Military Teaching Hospital laboratory, Saint-Mandé, within 48 hours of collection. Syndromic multiplex molecular gastrointestinal panel (Biofire FilmArray Gastrointestinal Panel, BioMérieux Laboratories, https://www.biomerieux-diagnostics.com/filmarray), which tests for 22 common gastrointestinal pathogens, including bacteria, viruses and protozoa, was used for syndromic diagnosis [20], according to manufacturer’s instructions. Genomic detection of the parasite in stool was confirmed by microscopic examination under 100x magnification of the stained stool samples using the Ziehl-Neelsen method for acid-fast staining, according to the technique described by Henriksen and Pohlenz [21]. In addition, a rapid lateral flow immunoassay for direct qualitative detection of *Cryptosporidium* spp. antigen (Cryptosporidium Xpect, Remel, Inc) was also performed according to manufacturer’s instructions.

### Environmental investigations

**Fig 1:**
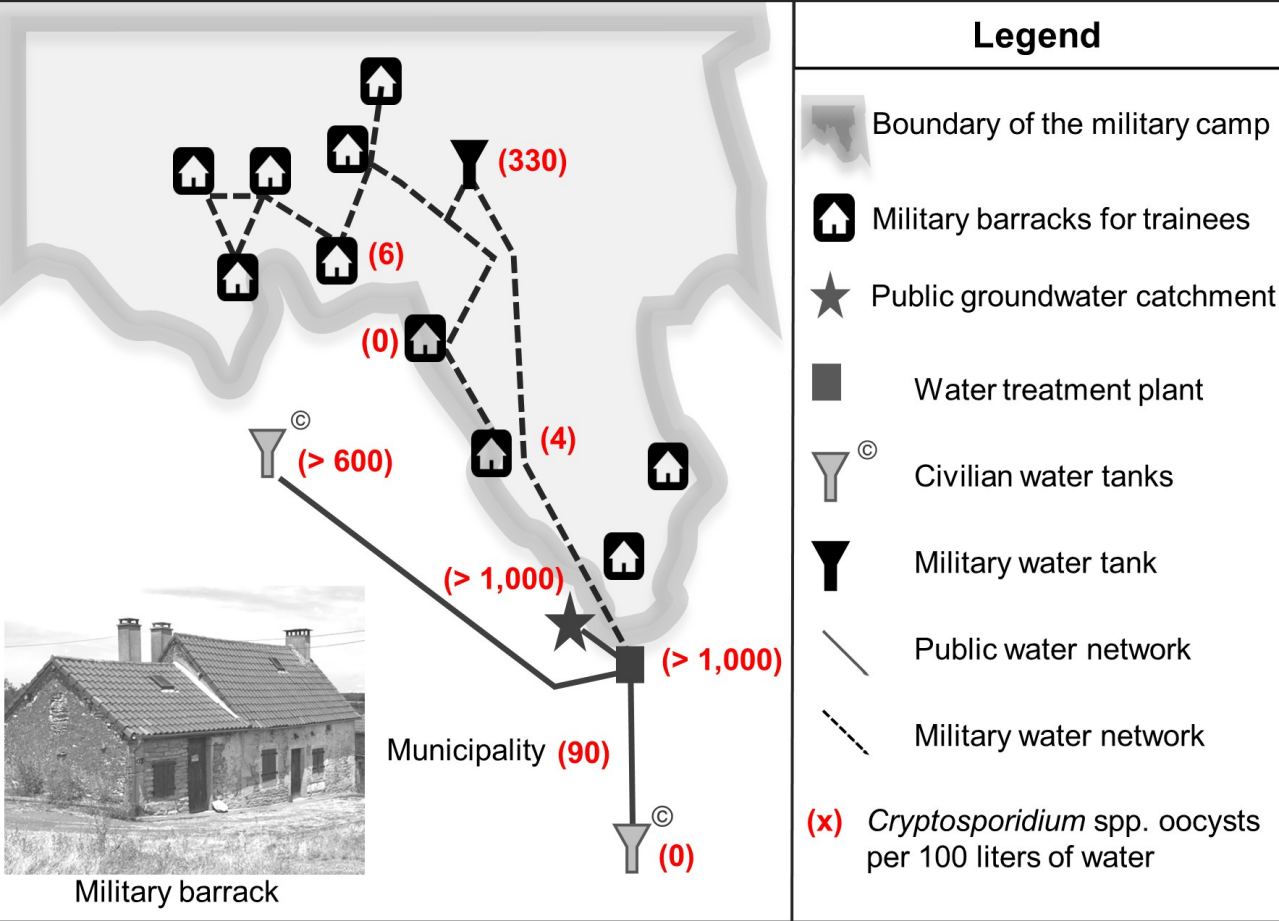
Cryptosporidiosis outbreaks, *Cryptosporidium* spp. oocysts in several water installations, France, 2017.

The military camp water network includes a water tower and a system of pipes that supply the main living area and the rustic barracks for trainees scattered around the camp (Fig 1). The camp is connected to the civilian water network, which is supplied by a groundwater catchment. The raw water is processed in a civilian water treatment plant. The treatment consists of direct sand filtration (i.e., without any pre-treatment using coagulation-flocculation process) followed by chlorination. Given the first results, which identified *Cryptosporidium* spp. in human stool, water samples (100 L—per sample) were collected for analysis at 10 points of the military and civilian water installations, from points of use connected to the military camp water system, including taps and the water tower, to the resource (Fig 1). The stages of collecting, transporting and storing the water samples prior to analysis were entirely managed by the laboratory in charge of water analysis, which is accredited according to the ISO 17025 standard for the performance of microbiological, physical and chemical testing on drinking water [22]. Firstly, the water samples were tested according to a program of regulatory analyses routinely carried out on the taps of the public drinking water network [23]. Details of the analytical methods used on the water samples are given in Table 1. The samples were then specifically tested for the parasites *Cryptosporidium* spp. and *Giardia* spp. using the French NF T90-455 standard method for sampling and enumeration of *Cryptosporidium* oocysts and *Giardia* cysts in water samples [24].

**Table 1:** Analysis parameters and testing methods applied to water samples, France, 2017.

Water samples testing		
	Analysis parameters	Analysis method
**Microbiological parameters**	• Spores of sulphite-reducing micro-organisms • Coliform bacteria • Intestinal Enterococci • *E*. *coli*	NF EN ISO 26461–2 standard NF EN ISO 9308–1 standard NF EN ISO 7899–1 standard NF EN ISO 9308–3 standard
**Physical parameters**	• Appearance, colour, smell, taste • Temperature	Internal method IEA03 electrical measurement
**Chemical parameters**	• Turbidity • Conductivity • pH • Free chlorine • Total chlorine	NF EN ISO 7027 standard NF EN ISO 27888 standard NF EN ISO 10523 standard NF EN ISO 7393–2 standard NF EN ISO 7393–2 standard

Corrective actions were rapidly implemented in order to reduce human exposure to *Cryptosporidium* spp. and to bring the production and distribution facilities of drinking water back into compliance. The main actions carried were: i/ restrictions on the use of water from the contaminated network (civilian and military populations) and implementation of a palliative supply of bottled water; ii/ installation of a temporary mobile ultrafiltration unit at the outlet of the resource treatment plant, allowing effective treatment of *Cryptosporidium* spp. and iii/ purging and disinfection of drinking water installations and release control analyses before lifting tap water use restrictions. At the same time a search for potential human and animal sources of contamination was carried out in order to understand and prevent further pollution of the groundwater.

### Species identification and strain genotyping

Positive stool and water samples (water filtration concentrates) for parasite detection were sent to the Cryptosporidiosis National Reference Centre Expert Laboratory (CNR-LE-Cryptosporidiosis) for cryptosporidiosis analysis (Rouen University Hospital, France). DNA was extracted from samples using the QIA Amp DNA Power Fecal kit (Qiagen) according to the manufacturer’s instructions. The identification of *Cryptosporidium* species was performed using real-time 18S SSU rRNA PCR and confirmed by GP60 sequencing as described [25,26].

### Ethical statement

Each patient received care adapted to their state of health provided by the French Armed Forces Health Service. All participants received information about the investigation and the disease, gave their consent and participated voluntarily. According to French regulations, as this was a severe outbreak with immediate public health threat, no ethical approval was required.

## Results

### Epidemiological investigations

**Fig 2:**
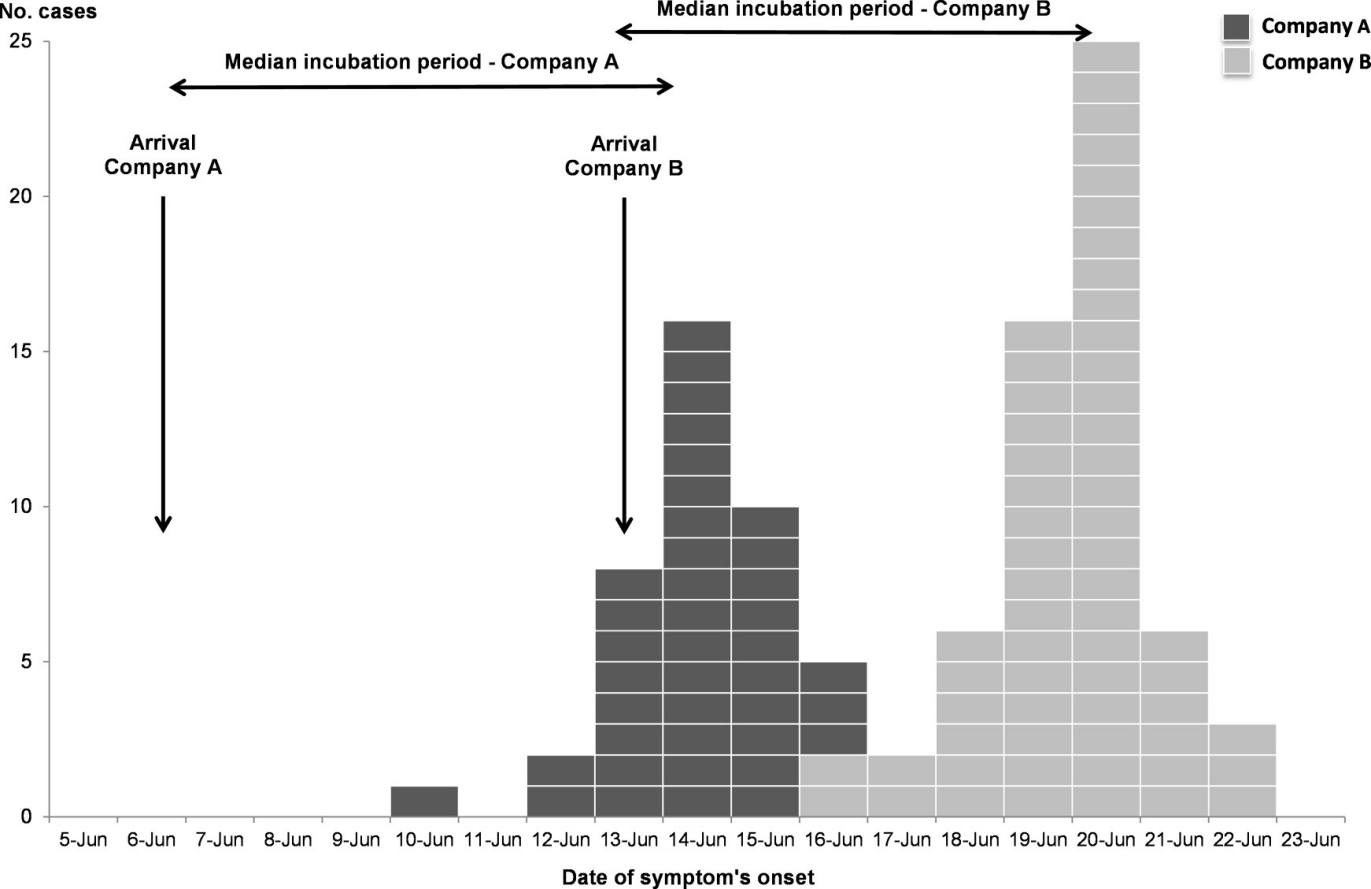
Cryptosporidiosis outbreaks in two companies of the French Armed Forces, epidemic curve, France, 2017, (N = 100 cases).

The two companies arrived separately in time and were independent populations (present in the camp at two different times and without common activities). The outbreaks started a few days after their arrival (Fig 2).

One hundred cases among a total of 360 trainees were identified, 40 in Company A and 60 in Company B resulting in a global attack rate of 27.8%. There were no AG cases among the permanent support staff of the military camp. The two successive outbreaks suggested a common and persistent source of exposure. Assuming the possibility of exposure to the pathogen on arrival at the camp, the median incubation time was estimated at eight and nine days for Company A and B respectively (Fig 2). Complete clinical data were available for 87 of the 100 cases with the following symptoms reported: abdominal pain (84%), diarrhoea (68%), nausea (53%), fever or feeling feverish (46%), and vomiting (26%). Symptoms lasted about a week for most of the cases and did not result in any severe case or hospitalization.

The case-control study in Company B was not conclusive. Foods were mostly based on combat rations, which are controlled and safe ready-to-eat canned meals. No statistically significant association was found between water consumption and disease, as both cases and controls consumed tap water from the drinking water installations of the military camp during training. We did not observe a dose-effect related to the amount of water daily consumed (S1 Table).

No AG outbreak was reported by the local health authorities in June 2017, among the 1,500 civilians supplied by the same water source. In addition, retrospective analysis of drug reimbursement data from 2015 to 2017 did not identify any AG cluster.

From May 30th to June 8th, the area received 55 mm of rainfall, for a mean expected value of 65 mm for the whole of June.7

### Microbiological investigations

*Cryptosporidium* spp. was first identified with syndromic multiplex PCR in 91% (10/11) and 100% (3/3) of stool samples from companies A and B respectively (Table 2). The presence of *Cryptosporidium* spp. oocysts was confirmed by direct microscopic examination. All the isolates were identified as *C*. *hominis* subtype IbA10G2, confirming that the same strain was involved in both outbreaks. Enteropathogenic *Escherichia coli*, *Campylobacter* spp. and *Clostridium difficile* were also found by multiplex PCR in three stool samples and considered as pathogen carriage in this context.

**Table 2:** Cryptosporidiosis outbreak, molecular examination of stool samples, France, 2017.

Molecular examinations									
	Syndromic multiplex PCR panel ^a^			18S SSU rRNA RT-PCR			GP60 sequencing		
Stool samples	Pathogens ^b^	n	%	Species	n	%	Subtype	n	%
Company A	*Cryptosporidium* spp.	10	91	*C*. *hominis*	10	100	IbA10G2	10	100
N = 11	EPEC^c^	1	9	-	-	-	-	-	
	*Campylobacter* spp.	1	9	-	-	-	-	-	-
Company B	*Cryptosporidium* spp.	3	100	*C*. *hominis*	3	100	IbA10G2	3	100
N = 3	*Clostridium difficile*	1	33	-	-	-	-	-	-

^a^ Biofire FilmArray Gastrointestinal Panel, BioMérieux

^b^ Co-infections were found in two cases

^c^ Enteropathogenic *Escherichia coli*

### Environmental investigations

Local laboratory testing confirmed the contamination of the entire water network with *Cryptosporidium* spp. Low (4 to 6 oocysts per 100 L) to moderate levels (330 oocysts per 100 L) of contamination were observed respectively in the taps of two dwellings and in the water tower in the military camp (Table 3, Fig 1). The highest levels of contamination (>1,000 oocysts per 100 L) were found in groundwater and in the water leaving the treatment plant. Groundwater was also contaminated with faecal bacteria and *Giardia* spp. Oocysts were found in the tap water of a house in the municipality (90 per 100 L), confirming the civilian population exposure. The same isolate *C*. *hominis*, subtype IbA10G2, was identified in the water samples collected on civilian and military drinking water installations and was the same as the one found in stool’s samples. Physicochemical parameters and chlorine concentration (≥ 0.3 mg/L in water storage facilities; ≥ 0.1 mg/L in tap water) were within the expected range.

**Table 3:** Cryptosporidiosis outbreak, laboratory testing of water samples, France, 2017.

Laboratory testing results				
	Bacteria	Enumeration (CFU ^a^ / 100 mL)	Parasites (Species & subtype)	Enumeration (oocysts / 100 L)
**Military network**				
Dwelling tap 1	Spores of sulphite-reducing micro-organisms Coliform bacteria Intestinal Enterococci *E*. *coli*	Absence	*Cryptosporidium* spp. (*C*. *hominis* IbA10G2)	4
Dwelling tap 2		Absence	*Cryptosporidium* spp. (*C*. *hominis* IbA10G2)	6
Dwelling tap 3		Absence	*Cryptosporidium* spp. (Na)^b^	Absence
Water fountain		Absence	*Cryptosporidium* spp. (Na)^b^	Absence
Water tank		Absence	*Cryptosporidium* spp.	330
**Civilian network**				
Dwelling tap	Spores of sulphite-reducing micro-organisms Coliform bacteria Intestinal Enterococci *E*. *coli*	Absence	*Cryptosporidium* spp. (Na)^b^	90
Water tank 1		Absence	*Cryptosporidium* spp. (Na)^b^	Absence
Water tank 2		Absence	*Cryptosporidium* spp. (*C*. *hominis* IbA10G2)	> 600
Treatment plant		Absence	*Cryptosporidium* spp. (*C*. *hominis* IbA10G2)	> 1,000
**Common water resource for military and civilian installations**				
	Intestinal Enterococci	289	*Cryptosporidium* spp. (*C*. *hominis* IbA10G2)	> 1,000
	*E*. *coli*	327		
	Spores of sulphite-reducing micro-organisms	Absence	*Giardia* spp. (Na)^b^	4

^a^ CFU: colony-forming units–

^b^ Na: Not assessed

As soon as the positive water test results for *Cryptosporidium* spp. were obtained, water restrictions (a ban on use of water for drinking and food preparation) were immediately implemented in the military camp and the municipality. As an emergency measure, bottled water was distributed to the entire population (civilian and military). An additional ultrafiltration module was installed at the water treatment plant one month after the second outbreak. A reinforced water quality monitoring program was implemented, consisting in a monthly analysis for *Cryptosporidium* spp. and *Giardia* spp. on the groundwater and at the outlet of the filtration treatment. The ultrafiltration module proved immediately to be very effective, as no *Cryptosporidium* spp. oocyst were found in the water after treatment. The civil health authorities decided to lift the water restrictions for the civilian population, after purging and disinfecting the water supply network. The same procedures were then carried out in a second phase on the water supply network in the military camp. This water treatment was secondarily completed by an ultraviolet disinfection system.

**Fig 3:**
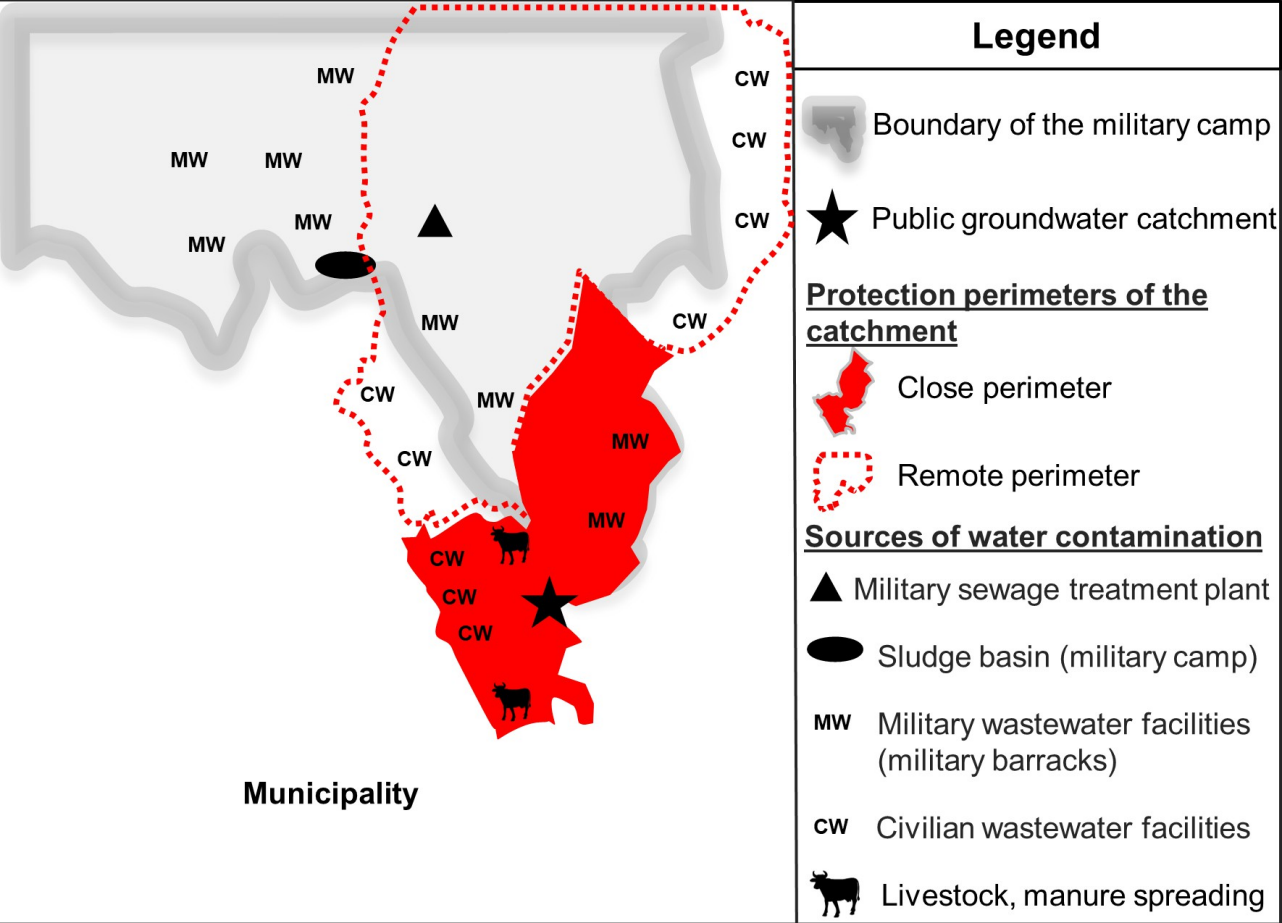
Cryptosporidiosis outbreaks, potential sources of contamination of the water resource (catchment), France, 2017.

Several activities were identified as potential sources of microbiological contamination of the ground water in and outside the military camp: wastewater treatment plant (WWTP), the lagoon and sludge spreading areas of the WWTP, the collection and disposal facilities for military dwelling wastewater, livestock grazing, livestock manure management (slurry pits, land application) and septic tanks (Fig 3). Polluting activities within the protection perimeters of the water resource were strongly restricted. The main restrictions concerned the ban on spreading sludge from the military camp’s wastewater treatment plant, restrictions on cattle grazing within the protection perimeters of the water resource and verification of the watertightness and strict monitoring of the frequency of emptying septic tanks. On the military camp, these actions were monitored by the local military command, under the technical supervision of the territorially competent army veterinary structure. The implementation of these restrictions was followed by the end of groundwater contamination. No further AG outbreak has been reported since in the FAF.

## Discussion

The occurrence of gastroenteritis among trainees had become so frequent in this military camp that the French service members had named it “Caylusite” (i.e., Caylusitis in English, in reference to the municipality near the camp). After successive and unexplained AG outbreaks this investigation identified the same *C*. *hominis* IbA10G2 isolate in the stools of patients and water samples, confirming a waterborne disease outbreak. In retrospect, these results suggest that the recurrent AG outbreaks recorded in the military camp were most likely waterborne disease outbreaks and caused by the same agent. The strain identified has already been involved in several cryptosporidiosis outbreaks [14, 15, 27].

Previous hydrogeological studies showed that the natural hydrogeological protection barrier of the water resource was very permeable, due to karstic rocks with faults [28]. The groundwater quality was therefore strongly influenced by the surface water. The underlying assumptions to explain the groundwater contamination were: i/ a polluting activity on the surface and ii/ direct infiltration into the groundwater during heavy rainfall. Indeed, one week before the first outbreak in June, the area received more than half of its normal seasonal rainfall. Similar occurrences have already been observed in other karst regions [29, 30]. Multiple sources of contamination were identified, including livestock grazing on the military camp, which could act as a reservoir for *C*. *hominis* [31]. However, because the water contamination ended several weeks after the reinforcement of the protection perimeter of the water resource, this was not investigated.

In case of suspected FBDO in the French Armed Forces, epidemiological, environmental and microbiological investigations are systematically performed [32, 33]. However, the pathogen involved is rarely identified, only one out of three over the period 1999 to 2013 [34]. The limitations identified to explain these poor results were the frequent absence of stool samples from patients and the fact that the parameters tested on stool samples are mainly targeted at bacterial agents. The search for *Cryptosporidium* spp. and other parasites in stool or water samples is not routinely performed by laboratories, especially in the absence of dedicated national guidance on testing [16]. This could explain why no causative pathogen was identified in previous outbreaks in this military camp. In order to better assess the pathogens involved in FBDO in the FAF, systematic stool sampling and multiplex PCR were implemented. Our findings validate the need for routine use of syndromic diagnosis in the investigation of AG [20]. However, multiplex PCR is very sensitive, and identification of carriage of other pathogens is possible. This highlights the importance of collecting stool samples from several patients to obtain matching results. In our study, the number of samples was limited but sufficient to conclude to an outbreak of cryptosporidiosis.

As expected, chlorination and sand filtration (cut-off threshold estimated at 10 μm) were not sufficient to effectively treat *Cryptosporidium* spp. (oocyst diameter is estimated to be between 3 to 5 μm) [5]. The use of an ultrafiltration process at the water treatment plant proved effective (absence of parasites), making it possible to lift the water restrictions. Regulatory analysis programs routinely implemented to monitor the quality of drinking water use for microbiological testing fecal indicator bacteria (FIB). The FIB mainly used in French regulatory surveillance programs of drinking water are *E*. *coli* and *Enterococcus* spp. [23]. However, these FIB do not correlate well with the presence of other pathogens like viruses and protozoa [35]. Firstly, because FIB can replicate in the environment without a host, unlike viruses or protozoa. Then, viruses and protozoa are more tolerant to chlorination than FIB. The microscopy-based reference method currently used to detect *Cryptosporidium* spp. in water is suboptimal because it requires a large volume of water [24]. This method of analysis cannot be used in routine to carry out drinking water quality monitoring. This should enhance developing new techniques for the detection of cryptosporidium in drinking water, in order to control of the parasite hazard at the different stages of the drinking water supply chain. Over the last few decades, various protocols based on molecular methods have been developed to improve the detection of *Cryptosporidium* spp. and *Giardia* spp. in aquatic samples. These techniques are more sensitive and would require less water sample volume than the microscopic examination [36].

The trainees passing through the military camp were probably immunologically naive against *C*. *hominis*, subtype IbA10G2. On the other hand, the civilian population of the nearby villages and the military camp permanent staff, may have been regularly exposed to the pathogen (civilian and military water facilities were supplied by the same parasite-contaminated water resource) leading to the acquisition of partial immunity [12]. This hypothesis could not be confirmed because no investigation was conducted by the civil health authorities among the population living in the Caylus area. But this would explain the absence of cases reported among them. Thus, military trainees, who were not inhabitants of the municipality of Caylus, served as sentinels, helping to highlight the contamination of civilian and military water facilities and, in retrospect, the exposure of the local civilian population to *Cryptosporidium* spp.

## Conclusions

Due to the absence of mandatory reporting, epidemiological data on cryptosporidiosis in the French general population are limited. These outbreaks focus attention on the lack of understanding of the epidemiology and prevention of this disease in France. Our study demonstrates the value of syndromic diagnosis during AG outbreak investigation. This method is now systematically used for stool testing during suspected FBDO in the FAF. Our results also highlight the importance of better assessing the microbiological risks associated with raw water and the need for sensitive and easy-to-implement tools for parasite detection. Furthermore, this type of investigation cannot be successful without a strong and multidisciplinary collaboration involving physicians, biologists, epidemiologists, and food/water safety specialists.

## Supporting information

S1 Table Cryptosporidiosis outbreak, case-control study, univariate analysis, France, 2017 (N = 101, 60 cases and 41 controls). (PDF)
