# Supplementary material for: Cryptosporidiosis outbreaks linked to the public water supply in a military camp, France
Source: PLoS Negl Trop Dis. 2022 Sep 12;16(9):e0010776. doi: 10.1371/journal.pntd.0010776 (PMC9499286; doi:10.1371/journal.pntd.0010776)
Supplement: S1 Table — (PDF) [file pntd.0010776.s001.pdf]

S1 Table. Cryptosporidiosis outbreak, case-control study, univariate analysis, France, 2017 (N=101, 60 cases and 41 controls).

	Cases (n=60)		Controls (n=41)		OR	P
	n	%	n	%	[95% CI]	
Food consumption						
Only combat ration [a]	16	26.7	5	12.2	ref.	0.15
At least one regular meal [b]	23	38.3	22	53.7	0.3 [0.1-1.0]	
Combat ration and other food [c]	21	35.0	14	34.1	0.5 [0.1-1.6]	
Water consumption						
Only bottled water	0		1	2.4	-	0.41
Bottled and tap water	60	100.0	40	97.6	-	
Daily water consumption						
<1L	8	13.3	8	19.5	ref.	0.53
1 to 3L	38	63.3	26	63.4	1.7 [0.5-5.2]	
>3L	14	23.3	7	17.1	2.2 [0.6-8.9]	

[a] Combat rations are controlled and safe ready-to-eat canned meals for service members in the field.
[b] The staff of the company had meals delivered.
[c] Foods such as crisps or biscuits. Pizzas were also consumed on the evening of arrival.
